# Supplementary material for: Ni-Catalyzed Enantioselective Intramolecular Mizoroki–Heck Reaction for the Synthesis of Phenanthridinone Derivatives
Source: J Org Chem. 2023 Jun 15;88(13):8203–26. doi: 10.1021/acs.joc.3c00202 (PMC10337041; doi:10.1021/acs.joc.3c00202)

DR-112-100 F7-8
PROTON CDCl3 {D:\nmrusers\malachowski} BM 23

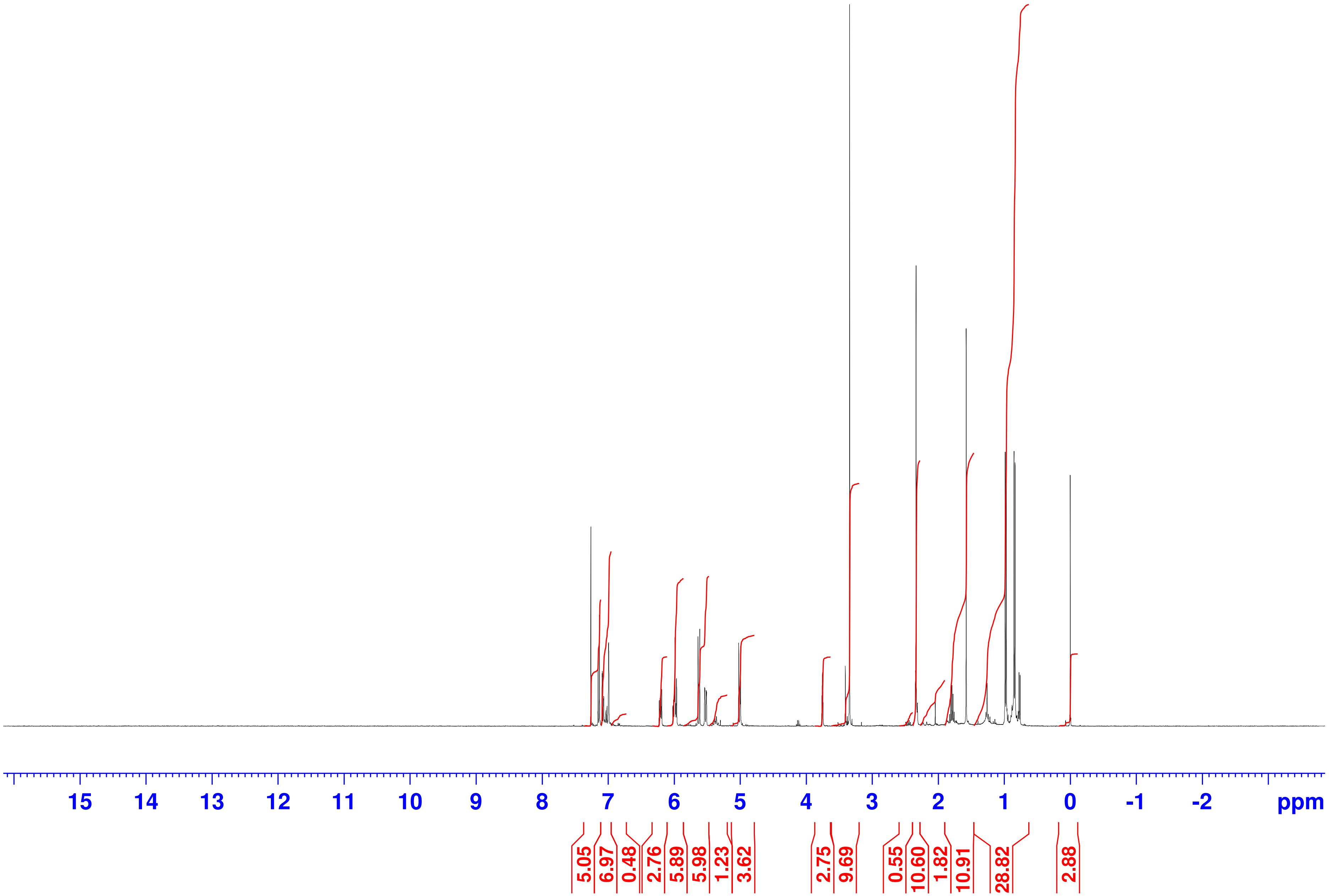

Supplement: Supplementary file 2 — jo3c00202_si_002.zip [file jo3c00202_si_002.zip › Heck products/2k iPr-NMOM-2Me Heck/1H iPr-NMOM-2Me Heck 2k/1/pdata/1/email_DR-112-100 F7-8_1_1.pdf]
